# High Mobility Group Proteins in Sepsis

**DOI:** 10.3389/fimmu.2022.911152

**Published:** 2022-06-02

**Authors:** Guibin Liang, Zhihui He

**Affiliations:** Department of Critical Care Medicine, The Third Xiangya Hospital, Central South University, Changsha, China

**Keywords:** high mobility group, protein, sepsis, non-coding RNAs, programmed cell death, drugs

## Abstract

Sepsis, a systemic inflammatory response disease, is the most severe complication of infection and a deadly disease. High mobility group proteins (HMGs) are non-histone nuclear proteins binding nucleosomes and regulate chromosome architecture and gene transcription, which act as a potent pro-inflammatory cytokine involved in the delayed endotoxin lethality and systemic inflammatory response. HMGs increase in serum and tissues during infection, especially in sepsis. A growing number of studies have demonstrated HMGs are not only cytokines which can mediate inflammation, but also potential therapeutic targets in sepsis. To reduce sepsis-related mortality, a better understanding of HMGs is essential. In this review, we described the structure and function of HMGs, summarized the definition, epidemiology and pathophysiology of sepsis, and discussed the HMGs-related mechanisms in sepsis from the perspectives of non-coding RNAs (microRNA, long non-coding RNA, circular RNA), programmed cell death (apoptosis, necroptosis and pyroptosis), drugs and other pathophysiological aspects to provide new targets and ideas for the diagnosis and treatment of sepsis.

## 1 Introduction

Sepsis is a syndrome caused by maladjusted body response to infection with no effective treatment (1). Globally, it’s estimated that there are 31.5 million sepsis patients and potentially 5.3 million mortalities every year, hospital mortality rates for general and severe sepsis in high-income countries were 17% and 26% respectively (2). It is estimated that the annual medical cost of 230,000 patients with sepsis treated in the ICU is about 4.6 billion US dollars, and the medical and social burden is heavy (3). The pathogenesis of sepsis is very complex, involving infection, inflammation, immune, blood coagulation, dysfunction of vascular endothelium cells (ECs) and tissue damage (4). The early stage of sepsis is characterized by the excessive systemic inflammatory immune response, and the late stage of sepsis is characterized by continuous immunosuppression (5). These processes may lead to cell dysfunction and eventually organ failure (6).

**Figure 1:**
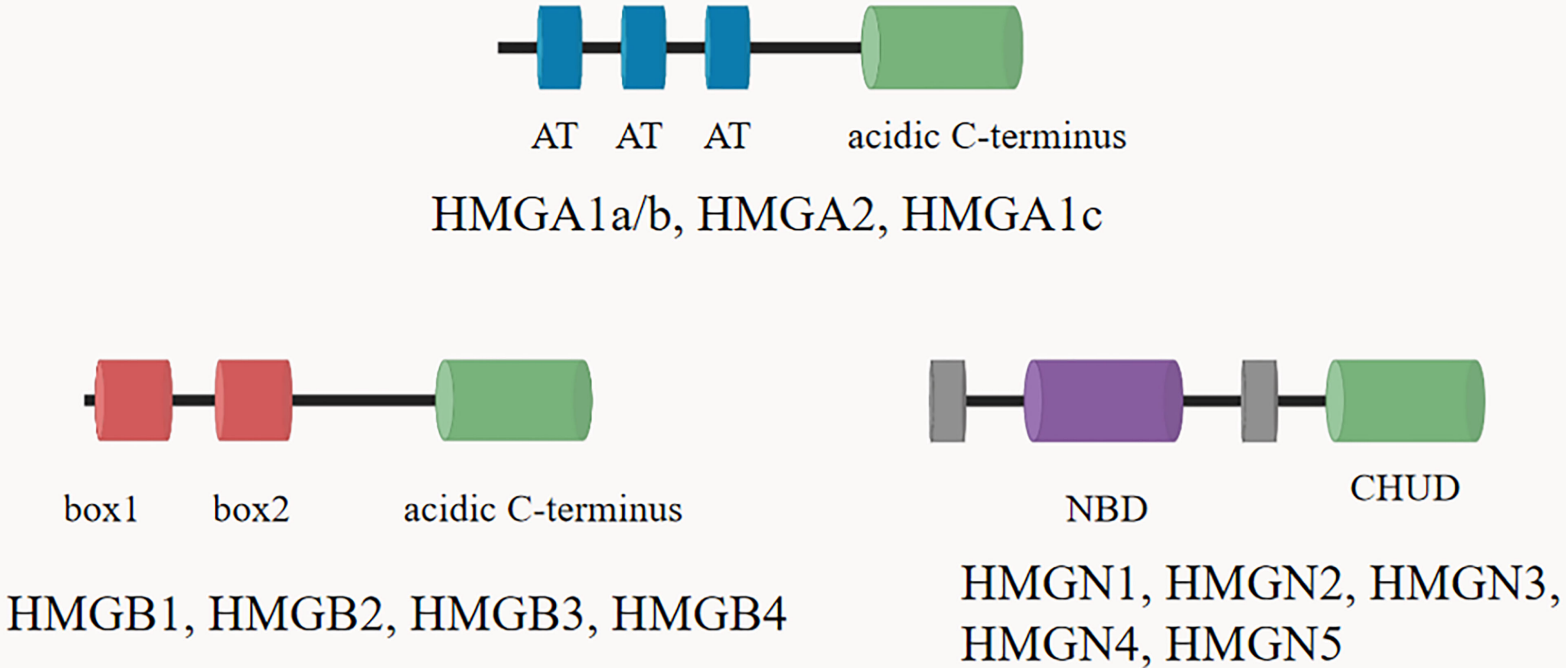
The structural features of the HMGs. Family members are listed above each diagram. All HMGAs contain three AT hooks and an acidic C-terminal part, except HMGA1c, which contains only two hooks. Each of the three members of the HMGB family contains two HMG boxes and an extended acidic C-terminus. The HMGN proteins are characterized by a positively charged nucleosomal binding domain (NBD) and a negatively charged C-terminal region named chromatin unfolding domain (CHUD).

High mobility group proteins (HMGs) are isolated from mammalian nuclei firstly and named according to their electrophoretic mobility (7). HMGs are the most abundant nuclear proteins except histones, and are widely expressed in tissues and organs (8). HMGs can bind distorted DNA uniquely and play a key role in transcription, recombination and DNA repair (9). HMGs are implicated in the pathogenesis of multiple diseases, including traumatic shock, infection, cancer, diabetes and autoimmune diseases (10–12). According to the structural and biological characteristics of different proteins, the HMGs are divided into three groups: HMGA, HMGB, and HMGN proteins (13). Each group of HMGs is characterized by a distinguished functional sequence motif: HMGA with “AT-hook”, HMGB with “HMG-box” and HMGN with “nucleosome binding domain (NBD)” (14) (**Figure 1**). Through these functional motifs, HMGs bind to specific structures in DNA or chromatin. HMGs are important inflammatory mediators in the fatal systemic reaction of sepsis, affect genomic functions not only by directly binding to chromatin but also by interacting with regulatory factors that affect gene expression.

Although the pathophysiology of sepsis is now much better understood, the search for pharmacotherapies for modulating the septic response has been unsuccessful, and the incidence and mortality of sepsis have not significantly decreased over the past two decades (15). More than 80 phase II and III clinical randomized controlled trials of drug therapy on sepsis have emerged around the world. In these trials, only a few drugs showed positive effect on the increase of survival rate in patients with sepsis. However, Chinese herbs spark interest in their use as a potential therapeutic agent. A summary of the linkage between HMGs and drug therapy in sepsis is necessary.

In this review, we summarized the roles of HMGs in sepsis. Understanding the role of HMGs in the pathogenesis of sepsis may help to propose potential effective treatment strategies, shorten the course of sepsis and improve the prognosis and survival rate of sepsis patients.

## 2 High Mobility Group Proteins

### 2.1 HMGA

The typical DNA-binding domain of HMGA is a palindrome amino motif, which preferentially binds to small grooves of short fragments of A/T-rich B-form DNA by recognizing structures rather than nucleotide sequences (16). There are two HMGA protein subgroups in mammals, HMGA1 and HMGA2. The HMGA1 subgroup consists of three proteins (HMGA1a, HMGA1b, and HMGA1c) (7).

#### 2.1.1 HMGA1

HMGA1 gene is located on human chromosome 6p21 (17). The HMGA protein family includes HMGA1a (107 amino acids, 11.7 kDa), HMGA1b (96 amino acids, 10.6 kDa) and HMGA1c (179 amino acids, 19.7 kDa) (18). HMGA1 plays an important role in chromatin change, chromatin remodeling, regulating gene transcription and DNA replication. HMGA1 regulates gene expression and transcription, improves the ability of cell proliferation and invasion and makes gene overexpressed, which will lead to the deterioration of cell proliferation and promote the formation of tumors. Moreover, HMGA1 plays an important role in the pathogenesis and treatment of sepsis (19–21).

#### 2.1.2 HMGA2

Human HMGA2 gene is composed of 5 exons and 4 introns, located in 12q13-15 (22). Most of its expression is located in the nucleus and plays a crucial role in regulating gene expression (22). It contains three independent DNA binding regions and an acidic hydroxyl terminal (22). Its amino acid composition is usually characterized by rich proline, basic amino acids and acidic amino acids, showing a high phosphorylation state (23). There are two phosphorylation sites on both sides of “AT-hook”. In S phase and G2/M phase, Ser can be phosphorylated by cyclin or cyclin-dependent kinase, which further affects its affinity with DNA or selectively regulates nuclear input (23). HMGA2 connects its AT-hook’ with specific DNA sequence to change the spatial conformation of its chromatin, further stretch, bend, loop or unzip its structure, and then regulate the transcription process of target gene (23). Its C-terminal can bind to H1 histone, affecting the conformation of nucleosome and participating in the formation of active chromatin. Therefore, HMGA2 is also known as structural transcription factor (24).

### 2.2 HMGB

HMGB is the most abundant protein in HMGs with molecular weight less than 30 kDa, and binds DNA specifically with DNA structure. In mammals, HMGB is highly conserved and contains four members, namely HMGB1, HMGB2, HMGB3 and HMGB4 (25). Although the protein encoded by HMGB has about 80% amino acid sequence homology, mice with knockout of HMGB1, HMGB2 or HMGB3 genes showed a recognizable phenotype (25). Each HMGB gene contains two DNA binding domains, named HMG box-A and HMG box-B. HMGB1-3 contain an acidic C-terminal, while HMGB4 lacks this acidic C-terminal (26). HMGB4 is a mammalian specific protein, which contains two HMG box domains, but lacks an acidic tail. Its nucleotide sequence is not conserved compared with other HMGB genes (27). HMG box in each HMGB can bind to DNA without any sequence specificity, to induce changes in DNA structure. HMG protein is widely expressed in all nucleated cells. The expression levels of HMGB1 and HMGB2 are the highest in immune cells among all kinds of cells. The expression of HMGB3 is relatively high in placenta, while the expression of HMGB4 is testicular specific (27).

#### 2.2.1 HMGB1

HMGB1 is the most studied member of HMGs. HMGB1 is encoded by the HMGB1 gene (13q12) in human beings (28). HMGB1 is the most expressed of HMGs family members and the most abundant component in all mammalian nuclei. It could be used as a construction factor to promote DNA folding and protein assembly on specific DNA targets (28). HMGB1 is the fastest migrating protein in the nucleus. It takes only 1-2s to cross from organelle to cell fluid (29). Because of its high mobility, HMGB1 could be found in cellular solutes such as mitochondria and lysosomes. HMGB1 protein has many biological functions in mammalian cells. Nuclear HMGB1 is involved in many DNA dependent biological activities, such as gene replication, repair, recombination, transcription and genome stability. HMGB1 plays an important role in cell growth, proliferation and death (30). When cells die or are about to die, HMGB1 is released into the extracellular space. Extracellular HMGB1 could act as damage-associated molecular patterns (DAMPs) to remind the immune system by recruiting inflammation, smooth muscle cells, vascular cells and stem cells (31). In addition, extracellular HMGB1 could act as an immune adjuvant to induce the activation or inhibition of cells (32–34). The activity of extracellular HMGB1 is regulated not only by the receptor, but also by its redox state and structure. In addition to the nuclear and extracellular roles of HMGB1, cytoplasmic HMGB1 could bind many proteins involved in autophagy, cancer evolution and unconventional secretory pathways (35).

**Figure 2:**
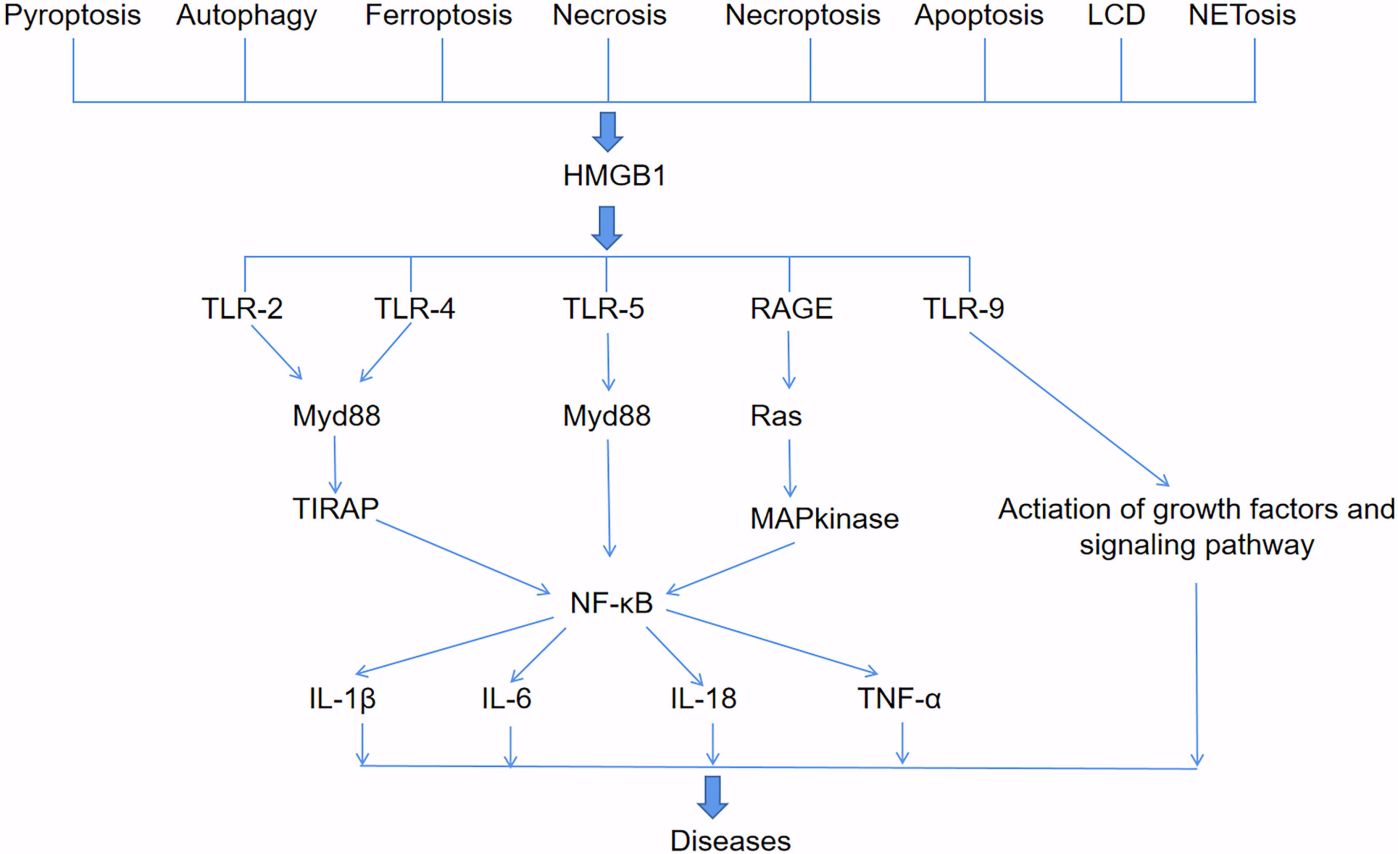
HMGB1-involved signaling pathways. HMGB1 can be passively released during various types of cell death, such as pyroptosis, autophagy, ferroptosis, necrosis, necroptosis, apoptosis, lysosome-dependent cell death (LCD) and NETosis. HMGB1 binds to TLR2, TLR4, TLR5, RAGE and TLR9 receptors to activate downstream pathways that produce pro-inflammatory cytokines such as IL-1β, IL-6, IL-18, and TNF-α, and lead to diseases.

HMGB1 involved in diseases and signaling pathways (**Figure 2**). HMGB1 can be passively released during various types of cell death, such as pyroptosis, autophagy, ferroptosis, necrosis, necroptosis, apoptosis, lysosome-dependent cell death (LCD) and NETosis. HMGB1 binding with toll-like receptors2 (TLR2), TLR4 and TLR5 receptors activates Myd88, which finally responsible for the activation of inflammatory transcription factor NF-κB by phosphorylation and ubiquitin mediated degradation of IκB (36). HMGB1 and mtDNA activate TLR9 signaling during hypoxia to induce tumor growth (37). HMGB1 binds to receptor for advanced glycation end-products (RAGE) receptors to activate Ras-oncogenes, leading to MAP kinase-mediated activation of NF-κB in inflammatory pathways (38). Moreover, activated NF-κB promotes the expression of different downstream pro-inflammatory cytokines such as IL-1β, IL-6, IL-18 and TNF-α, which is responsible for diseases progress (39). HMGB1 released by hepatocytes binds to LPS and is internalized into lysosomes of macrophages and endothelial cells through RAGE. Subsequently, HMGB1 permeates the phospholipid bilayer in the acidic environment of lysosomes. This causes LPS to leak into the cytoplasm and activate caspase-11. Loss of hepatocyte HMGB1, inhibition of hepatocyte HMGB1 release, neutralization of extracellular HMGB1 or RAGE deficiency can prevent caspase-11-dependent pyroptosis and death in endotoxemia and bacterial sepsis (40).

#### 2.2.2 HMGB2

The expression of HMGB2 is mainly limited to adult mouse lymphoid organs and testis (41). HMGB2 exists in all cultured cells and is abundant in thymus. Male HMGB2^-/-^ mice showed defects in spermatogenesis, which was caused by the degeneration of Sertoli cells and germ cells in seminiferous tubules and sperm immobility (42). The proinflammatory activity of extracellular HMGB2 was significantly lower than that of HMGB1 (43). In experimental and clinical acute lung injury (ALI), the content of extracellular HMGB2 increases, which means that HMGB2 has a potential role in tissue injury (43). Küchler R et al. identified competent antibacterial activity of HMGB2 against Escherichia coli, and demonstrated that the two DNA-binding domains (HMG boxes A and B) are crucial for the antibiotic function (44). Thought measure titers of anti-HMGB1/HMGB2 antibodies and anti-Saccharomyces cerevisiae antibodies (ASCA) in the sera of 213 patients with ulcerative colitis (UC) and 93 with Crohn’s disease (CD), using enzyme-linked immunosorbent assays. The presence of serum anti-HMGB2 antibody contributes to inflammatory bowel disease, which indicates that extracellular HMGB2 plays a role in regulating autoimmunity (45).

#### 2.2.3 HMGB3

HMGB3 was first discovered by Marco Bianchi and his colleagues in 1998, it could be used as an expression sequence tag (EST) for preferential expression in embryonic tissues (46). HMGB3 protein was highly expressed in embryos (46), most lymphocytes and bone marrow progenitor cells. HMGB3 is related to B cell differentiation and hematopoietic stem cell self-renewal and proliferation (47, 48).

#### 2.2.4 HMGB4

HMGB4 was a mammalian specific protein discovered by Irwin Davidson in 2009, it could be used as a new member of mammalian HMGB family (27). The molecular weight of HMGB4 protein is about 21 kDa. It contains a pair of DNA domains of HMG box and lacks acidic tail. Its nucleotide sequence is more conservative than that of other HMGB family members (27). Compare with HMGB1, HMGB4 is usually a transcription inhibitor encoded by intron free genes. Similar to other HMGBs, HMGB4 has a potential role in tumorigenesis. Overexpression of HMGB4 could inhibit the proliferation of breast cancer cells (49). HMGB4 has a potential role in regulating the anticancer activity of cisplatin (50). Up to now, there is no report of HMGB4 in sepsis.

### 2.3 HMGN

The HMGN group includes five members: HMGN1, HMGN2, HMGN3, HMGN4 and HMGN5 (51). The molecular structure of this group includes three obvious functional domains, including bilateral nuclear localization signal (NLS), nucleosome binding domain (NBD) and regulation domain (RD). HMGN gene is mainly expressed in vertebrates and located on different chromosomes. The HMGN1 gene in human is located on 21q22, HMGN2 on 1p36.1, HMGN3 on 6q14, HMGN4 on 6p21 and HMGN5 on Xp13. HMGN protein could interact with nucleosome particles to participate in DNA replication, transcription, repair and recombination (52). Most studies on HNGNs focus on tumors (53–55).

#### 2.3.1 HMGN1

HMGN1 was first extracted from porcine thymus by Graham in 1977 (56). Then Johns isolated HMGN1 from chicken erythrocyte nuclei (57). Human HMGN1 protein is composed of 100 amino acids with a size of about 10.6 kDa (57). Its coding gene is located on human chromosome 21p22 and mouse chromosome 16 (57). In addition to binding to DNA and affecting its replication and transcription, HMGN1 also plays a role in DNA damage repair, organ differentiation and development, disease occurrence (57).

#### 2.3.2 HMGN2

HMGN2 is the most conservative member of HMGN group. Its molecular weight is about 9.2kDa and consists of 90 amino acid residues. HMGN2 coding gene is located on human chromosome 1p36 and mouse chromosome 4. It was first extracted from chicken red cell nucleus. HMGN2 has many functions, such as changing chromosome activity, participating in organ differentiation and development, regulating DNA damage repair (58).

#### 2.3.3 HMGN3

The structure of HMGN3 is highly conserved and contains two splice variants, HMGN3a and HMGN3b. HMGN3a has classical HMGN regions (HLS, NBD and RD), while HMGN3b lacks regions (30). HMGN3 coding gene is located on human chromosome 6p14 (30). Fan Y et al. identified HMGN3 as a marker gene for sepsis through bioinformatics analysis, but no clinical verification was carried out (59).

#### 2.3.4 HMGN4

HMGN4 was the least studied component in HMGN group. Birger Y et al. first found a transcript in the gene bank during data search, and then confirmed it as HMGN4 (60). Its coding gene is an intron free gene which located on human chromosome 6p21. HMGN4 exists only in primates and consists of 90 amino acids with a size of about 9.5 kDa (61).

#### 2.3.5 HMGN5

Shirakawa H et al. found HMGN5 by searching GenBank database in 2000 (62). King and Francomano in 2001 discovered the structure of HMGN5 protein-coding gene (63). HMGN5 gene is located on human chromosome Xp13. The protein is composed of 282 amino acids with a size of about 31.5 kDa (30). During embryonic development, the expression level of HMGN5 protein in different cells or tissues is very different (64).

## 3 HMGs in Sepsis

**Figure 3:**
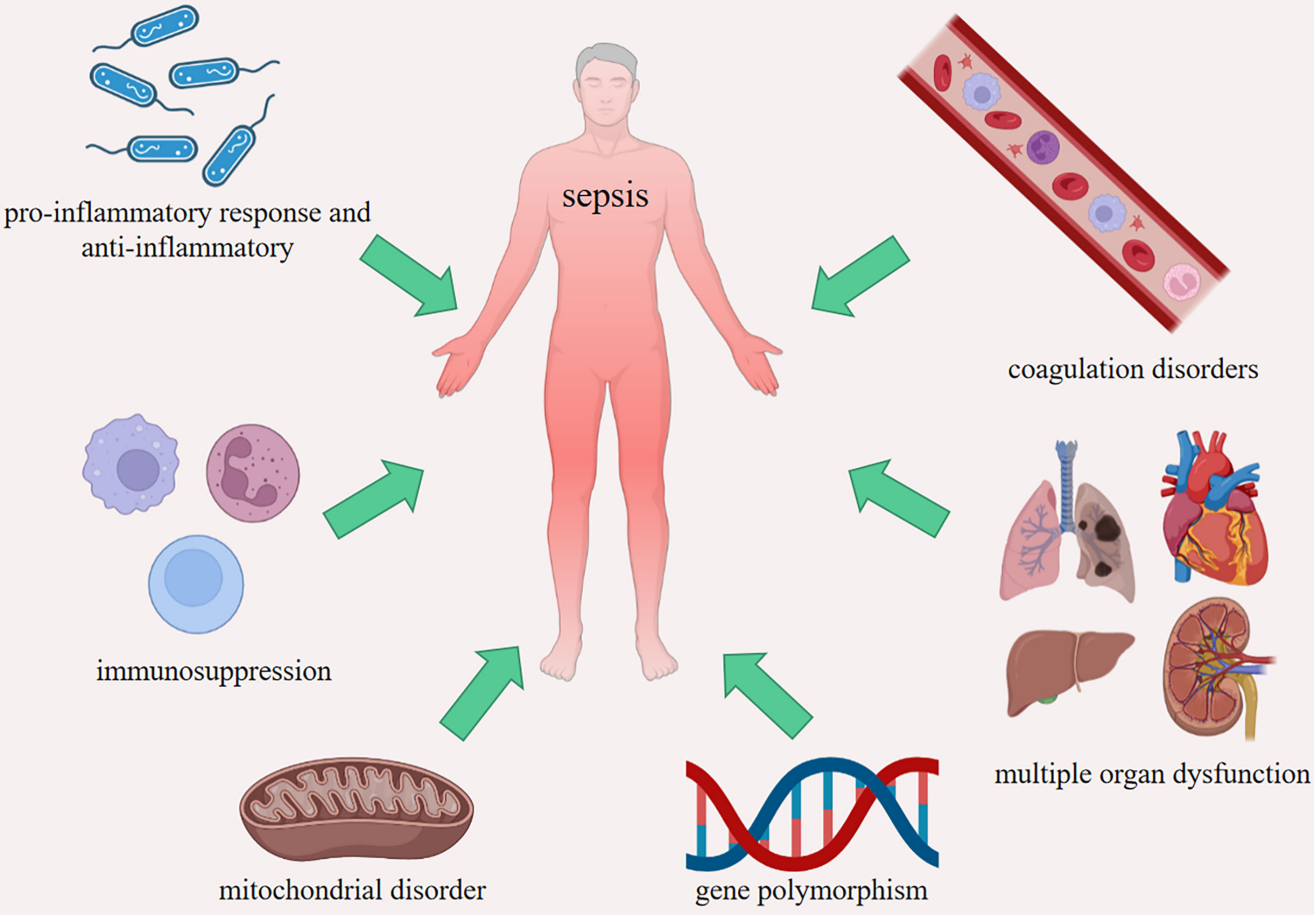
The fundamental pathogenesis of sepsis. Imbalance between pro-inflammatory response and anti-inflammatory, coagulation disorders, immunosuppression, multiple organ dysfunction, mitochondrial disorder and gene polymorphism.

The definition of Sepsis 3.0 was used since 2016, which includes patients with life-threatening organ dysfunction caused by an uncontrolled host response to infection, and sequential organ failure assessment (SOFA) score ≥ 2 (65). The fundamental pathogenesis of sepsis is not fully clear, including imbalance between pro-inflammation and anti-inflammation, coagulation disorders, immunosuppression, multiple organ dysfunction, mitochondrial disorder and gene polymorphism (**Figure 3**). HMGs participate in the occurrence and development of sepsis through most of the above mechanisms. After infection, the host immune system-mediated pro-inflammatory response will respond within a few hours. Previous studies have suggested that with the development of pro-inflammatory response, the body initiated compensatory anti-inflammatory response syndrome (CARS) (66). In fact, the process is more complex, and the pro-inflammatory reaction and anti-inflammatory reaction are more likely to occur simultaneously in the early stage (67, 68).

The researches regarding HMGs in sepsis involve many aspects, including cellular pathways, molecular mechanisms and potential drug screening (69–71). Most studies regarding HMGs in sepsis were of the interaction between HMGs and non-coding RNAs, programmed cell death and drugs, so we summarized the roles of HMGs in sepsis mainly from the aspects of non-coding RNAs (microRNA, long non-coding RNA, circular RNA), programmed cell death (apoptosis, necroptosis and pyroptosis), drugs and mechanism of sepsis so as to provide some new targets and ideas for the diagnosis and treatment of sepsis.

### 3.1 HMGs and Non-Coding RNAs in Sepsis

Non-coding RNA refers to RNA that does not encode protein, including microRNA (miRNA), long non-coding RNA (lncRNA) and circular RNA (circRNA) (72). Non-coding RNAs interact with proteins, DNA and RNA, participate in a variety of cellular activities, including gene activation, RNA splicing and protein translation (73). Structural features and function of miRNA, lncRNA and circRNA are shown in **Table S1**. Studies on HMGs and sepsis involving non-coding RNAs are shown in **Table 1**.

**Table 1:** Non-coding RNAs in HMGs and sepsis. Reference given as PMID.

Name	Non-coding RNAs	Programmed cell death	Disease	Study Model	Summary	Ref (PMID)
HMGA1	lncR-IGF2-AS	pyroptosis	sepsis	human	lncRNA IGF2-AS regulates nucleotide metabolism by mediating HMGA1 to promote pyroptosis of EPCs.	35082972
HMGA2	miR-98	/	sepsis-induced cardiac dysfunction, liver and lung injury	mice	miR-98 protects sepsis mice from cardiac dysfunction, liver and lung injury by negatively mediating HMGA2 *via* the inhibition of the NF-κB signaling pathway.	31234706
HMGB1	miR-25	/	sepsis	human	inhibiting expression of miR-25 may serve a role in up-regulating HMGB1 expression and resulting in sepsis.	30250569
HMGB1	miR-103a-3p	apoptosis	sepsis-induced liver injury	mice	miR-103a-3p could attenuates sepsis-induced liver injury by targeting HMGB1.	32556802
HMGB1	miR-103a-3p	apoptosis	sepsis	mice/macrophages	miR-103a-3p alleviates LPS-induced sepsis and MODS *in vitro* by decreasing HMGB1.	33333289
HMGB1	miR-129-5p	apoptosis	sepsis-induced AKI	podocytes/mice	overexpression of miR-129-5p protects against LPS-induced podocyte apoptosis, inflammation and AKI *in vivo* and *in vitro* models of sepsis.	33039954
HMGB1	miR-129-5p	apoptosis	sepsis-induced ALI	mice/(MLE)-12 cells	miR-129-5p protects against sepsis-induced ALI by decreasing HMGB1 expression.	32682121
HMGB1	miR-181-5p	/	sepsis	mice	miR-181-5p-mediated protective effects in septic mice are modulated, at least partially, through post-transcriptional repression of HMGB1 protein expression.	33015817
HMGB1	miR-193-3p	apoptosis	sepsis-induced myocardial injury	mice	miR-193-3p targets STAT3 expression to reduce HMGB1 expression, thereby reducing septic myocardial damage.	34503521
HMGB1	miR-205	apoptosis	sepsis-induced AKI	rat	miR-205 agonist could improve the pathological morphology in the sepsis rats with renal injury, improve renal cell apoptosis, and inhibit the protein levels of HMGB1 and PTEN in renal tissues.	31858563
HMGB1	miR-205-5b	/	sepsis	RAW264.7 cells	miR-205−5b inhibites HMGB1 expression in LPS-induced sepsis.	27246725
HMGB1	miR-212-3p	/	sepsis	macrophage cells	miR-212-3p directly targeted HMGB1 to suppress inflammatory response in LPS-induced cells.	27940320
HMGB1	miR-381-3p	apoptosis	sepsis-steered cardiac damage	rat	miR-381-3p restores the inflammatory response and myocardial dysfunction caused by sepsis *via* HMGB1.	34784841
HMGB1	lncR-NEAT1	apoptosis	sepsis-induced ALI	mice/alveolar epithelial A549 cell	lncR-NEAT1 may aggravate the progression of ALI and ARDS by inducing alveolar epithelial cell injury and inflammation *via* HMGB1/RAGE signaling.	32089649
HMGB1	lncR-HOX/miR-22	apoptosis	sepsis-induced kidney injury	rat	lncR-HOTAIR is up-regulated in sepsis-induced kidney injury, which promotes HK-2 cell apoptosis in kidney injury through the miR-22/HMGB1 pathway.	30130540
HMGB1	lncR-PVT1/miR-29a	/	sepsis-induced myocardial injury	mice	lncR-PVT1 promotes M1 polarization and aggravates LPS induced myocardial injury *via* miR-29a/HMGB1 axis.	33840587
HMGB1	lncR-MALAT1/miR-370-3p	apoptosis	sepsis-induced AKI	HK-2 cells	paclitaxel protects against LPS-induced AKI *via* the regulation of lnc-MALAT1/miR-370-3p/HMGB1 axis.	32998017
HMGB1	lncR-GAS5/miR-449b	/	sepsis-induced myocardial depression	mice	lncRNA GAS5 promotes sepsis-induced myocardial depression *via* the miR-449b/HMGB1 axis and the following NF-κB activation.	33645622
HMGB1	circR-TLK1/miR-17-5p	apoptosis	sepsis-induced cardiomyopathy	human cardiomyocytes/rat	circR-TLK1 sponges miR-17-5p to aggravate mtDNA oxidative damage, mitochondrial dysfunction and cardiomyocyte apoptosis *via* activating PARP1/HMGB1 axis during sepsis.	34410682
HMGB1	circR-TLK1/miR-106a-5p	apoptosis	sepsis-induced AKI	rat	circR TLK1 contributes to sepsis-associated AKI by regulating inflammation and oxidative stress through the miR-106a-5p/HMGB1 axis.	34250012
HMGB1	circR-PTK2/miR-181c-5p	apoptosis	sepsis-induced encephalopathy	mice/microglia	circR-PTK2-miR-181c-5p-HMGB1: a new regulatory pathway for microglia activation and hippocampal neuronal apoptosis induced by sepsis.	33952191
HMGB3	miR-128-3p	apoptosis	sepsis-induced ALI	rat	down-regulating SNHG16 alleviates the sepsis-mediated ALI by regulating miR-128-3p/HMGB3.	34092219

EPCs, endothelial progenitor cells; AKI ,acute kidney injury; HK2, Human renal tubular epithelial cell line; ALI, acute lung injury; MLE-12, murine alveolar epithelial cell line-12 cells; ARDS, acute Respiratory Distress Syndrome; SNHG16, small nucleolar RNA host gene 16.

/ means the study do not involve programmed cell death.

#### 3.1.1 miRNA and HMGs in Sepsis

miRNA is a kind of small single-stranded RNA molecule encoded by endogenous genes, with a length of about 22 nucleotides. It is not translated into protein, but participates in the process of protein translation (74). miRNA has three main characteristics: time and space specificity, high selectivity and conservation of structure and sequence, which makes it an ideal biomarker for medical clinical diagnosis (75). Compare with other biomarkers, miRNA remains stable and highly conservative in circulation (75). The existence of miRNA in body fluids resulted from multiple mechanisms: the release of miRNA from cell fragments after apoptosis or necroptosis, the active shedding of microcapsules (including exosomes), and the secretion of protein complex (76–78).

In sepsis patients, Zhu C et al. found that inhibiting the expression of miR-25 up-regulate the expression of HMGB1 and promote the secretion of inflammatory cytokines such as TNF-α and IL-6, resulting in sepsis (71). In sepsis mice model induced by cecum ligation and puncture (CLP), miR-103a-3p had a protection role in sepsis-induced liver injury by inhibiting the expression of HMGB1 (79). The rescue of miR-181-5p expression by lentivirus expression vector protects against sepsis-induced renal and hepatic dysfunction. miR-181-5p might function as an HMGB1 antagonist for alleviating sepsis-induced systemic inflammation (80). In sepsis rat model induced by CLP, Sun J et al. detected the expression profiles of SNHG16, miR-128-3p and HMGB3 by quantitative reverse transcription PCR and western blot. The levels of proinflammatory factors and proteins were detected by ELISA and western blot. The results showed that sepsis-mediated ALI was regulated by miR-128-3p/HMGB3 (81). miR-17-5p aggravated mtDNA oxidative damage, mitochondrial dysfunction and cardiomyocyte apoptosis during sepsis by activating HMGB1 (82). miR-181c-5p-HMGB1 signaling pathway could regulate sepsis-induced microglia activation and hippocampal neuron apoptosis (83). In another study, septic mice were injected with miR-193-3p agomir, miR-193-3p antagomir or siRNA-STAT3, and then the expressions of miR-193-3p, STAT3 and HMGB1 in myocardium of septic mice were detected. The researchers confirmed that miR193-3p reduced septic myocardial injury by reducing the expression of HMGB1 (84). In the cellular inflammation model, miR-103a-3p alleviated LPS-induced sepsis and multiple organ dysfunction syndrome (MODS) *in vitro* by reducing the expression of HMGB1 (85). LncRNA GAS5 could bind to miR-449b and improve the expression of HMGB1, thereby activating NF-κB signal, Gao H et al. found that miR-449b/HMGB1 axis could promote sepsis-induced myocardial inhibition (86). In sepsis mice model induced by CLP, the expression levels of miR-129-5p and HMGB1 were measured by quantitative real-time polymerase chain reaction and western blot, and the correlation between miR-129-5p and HMGB1 was verified by luciferase assay and RNA immunoprecipitation. The result indicated that miR-129-5p protected against sepsis-induced ALI by decreasing HMGB1 expression (87). miR-98 protected sepsis mice from cardiac dysfunction, liver and lung injury by negatively mediating HMGA2 (88). miR-205 influenced renal injury in sepsis rats through inhibiting HMGB1 (89). miR129-5p attenuated LPS-induced podocyte apoptosis, inflammation, and acute kidney injury (AKI) through HMGB1, and plays a protective role in sepsis models *in vivo* and *in vitro (* 90). Through estimating the effects of paclitaxel, lncRNA-MALAT1, miR-370-3p and HMGB1 on the proliferation and apoptosis of HK-2 cells injured by LPS, Xu L et al. found that knockdown of miR-370-3p inhibited the expression of HMGB1 (91). Overexpression of miR-381-3p could inhibit the mRNA expression of HMGB1, repress H9c2 cells apoptosis and inflammatory response, and stimulate and promote the vitality of H9c2 cells. The result indicated that miR-381-3p restored sepsis-induced inflammatory response and myocardial dysfunction through HMGB1 (92). Xu HP et al. discovered that circRNA TLK1 enhanced HMGB1 expression by sponging miR-106a-5p in the HK-2 cells, and miR-106a-5p and HMGB1 were involved in circTLK1-mediated injury of LPS-treated cells, miR-106a-5p/HMGB1 axis could regulate inflammation, oxidative stress and promote sepsis-related AKI (93). Overexpression of miR-212-3p inhibited the cytoplasmic translocation of HMGB1 in LPS-induced RAW264.7 cells, which indicated that miR-212-3p directly targeted HMGB1 to suppress inflammatory response in LPS-treated macrophage RAW264.7 cells (94). HMGB1 expression negatively correlated with miR−205−5b expression in LPS-induced sepsis. By contrast, HMGB1 expression in LPS-stimulated RAW264.7 cells was increased following transfection with miR−205−5b inhibitor, which indicated that miR-205−5b inhibited HMGB1 expression in LPS-induced sepsis (95). Altogether, these findings indicate that HMGs play important roles in sepsis by regulating miRNAs.

#### 3.1.2 LncRNA and HMGs in Sepsis

LncRNA is a kind of RNA transcribed by RNA polymerase II, with a length of more than 200 nucleotides and no clear protein-coding effect (96). The current gene coding database shows that there are about 17904 lncRNAs and 14739 pseudogenes in the human genome. According to the relative position between lncRNA and coding gene, it could be divided into sense lncRNA, antisense lncRNA, bidirectional lncRNA, intergenic lncRNA and intron lncRNA.

In human studies, as reported, lncRNA IGF2-AS binds HMGA1 to regulate nucleotide metabolism, promoting pyroptosis of endothelial progenitor cells (EPCs) in sepsis patients (21). In LPS-induced ALI mice models, lncRNA nuclear paraspeckle assembly transcript 1 (NEAT1) may induce alveolar epithelial cell injury and inflammation through HMGB1, thereby aggravating the progression of ALI and acute respiratory distress syndrome (ARDS) (97). Down-regulation of lncRNA PVT1 by miRNA-29a/HMGB1 axis attenuates the polarization of macrophage M1 and alleviates sepsis-induced myocardial injury (98). LncRNA GAS5 might through HMGB1 and subsequent NF-κB promote sepsis-induced myocardial inhibition (86). LncRNA HOTAIR promotes HK-2 cell apoptosis in sepsis-induced renal injury through HMGB1 (99). *In vitro*, paclitaxel could prevent LPS-induced AKI by regulating lncRNA MALAT1/miR-370-3p/HMGB1 axis, suggesting that paclitaxel may be used as a therapeutic drug to reduce sepsis-related AKI (91). These findings also suggest that HMG plays an important role in sepsis by regulating lncRNA. Up to now, there is no report about HMGN linked with lncRNA in sepsis.

#### 3.1.3 CircRNA and HMGs in Sepsis

CircRNA is a kind of non-coding RNA with single-stranded closed-loop structure (100). It was once considered as an intermediate or wrong by-product in the process of RNA splicing (101). Recent studies have confirmed that circRNA is a non-random product with extensive existence, rich expression, stability and conservation (102). CircRNA has become a research hotspot in recent years. circRNAs are closed long non-coding RNAs, in which the 5’ and 3’ termini are covalently linked by back-splicing of exons from a single pre-mRNA. There are three kinds of circRNA: exonic circRNA (ecircRNA), exonic-intronic circRNA (EIciRNA) and circular intronic RNA (ciRNA) (103). CircRNAs have great potential as a new diagnostic biomarker and target for treatment of various diseases, including type 2 diabetes, cancer and sepsis. In sepsis rat model induced by CLP, circRNA circTLK1 regulates inflammation and oxidative stress through HMGB1, resulting in sepsis-related AKI (93). Li M et al. found CircRNA hsa_circ_0008305 (circPTK2) regulates sepsis-induced microglia activation and hippocampal neuron apoptosis through HMGB1 (83). *In vitro* and *in vivo*, circR-TLK1 aggravated mtDNA oxidative damage, mitochondrial dysfunction and cardiomyocyte apoptosis during sepsis by activating HMGB1 (82). There are relatively few researches about CircRNA and HMGs in sepsis. This area needs to be explored further. Up to now, there is no report about HMGA or HMGN linked with circRNA in sepsis.

### 3.2 HMGs and Programmed Cell Death in Sepsis

Sepsis can cause programmed cell death, including apoptosis, necroptosis and pyroptosis. Moreover, cell death is essential for understanding the pathophysiological basis of sepsis. HMGs are considered as the leading triggers of programmed cell death (PCD). Many studies have provided sufficient evidence linking HMGs’ role in cell death and sepsis (40, 104–106). The study on HMGs and PCD in sepsis facilitates the development of effective prevention and treatment for sepsis. The characteristics of apoptosis, necroptosis and pyroptosis are shown in **Table S2**.

#### 3.2.1 Apoptosis and HMGs in Sepsis

Apoptosis is a physiological and active death process of cells under certain physiological or pathological conditions through the activation, expression and regulation of a series of genes. It plays an important role in the life process and the maintenance of body homeostasis. During the process of cell death, DNA fragments and other cell wastes arise after chromosome or chromatin pyknosis and rupture which produces apoptotic body. Apoptotic body is quickly swallowed by phagocytes, so as to avoid the diffusion of cell contents to the surrounding and subsequent inflammation (107). Therefore, there is no extravasation of cell contents and inflammatory reaction in apoptosis. To date, research indicated that there are four main apoptotic pathways: the extrinsic or death receptor pathway, the intrinsic or mitochondrial pathway, intrinsic endoplasmic reticulum pathway and perforin/granzyme pathway (108).

As a dynamic regulator of gene transcription and chromatin remodeling, HMGA1 played an important role in the pathological process of sepsis-induced cardiomyopathy. In sepsis-induced cardiomyopathy models induced by LPS, the overexpression of HMGA1 exacerbates the inflammation and apoptosis of cardiomyocytes, while the silencing of HMGA1 alleviates the inflammation, but aggregates the apoptosis of cardiomyocytes (109). In order to study the potential causal relationship between apoptosis and organ injury, Qin et al. used the broad-spectrum caspase inhibitor Z-VAD-FMK in septic mice. They found that Z-VAD-FMK treated septic mice had decreased levels of HMGB1 which inhibited macrophage apoptosis in the spleen and thymus (69). CircTLK1 aggravates mtDNA oxidative damage, mitochondrial dysfunction and cardiomyocyte apoptosis by activating HMGB1 during sepsis (82). miR-181b directly targets HMGB1, and down-regulating HMGB1 in sepsis could reduce inflammatory factors and myocardial injury and inhibit cardiomyocyte apoptosis (110). miR-22-3p is significantly down-regulated in a rat model of sepsis-induced AKI and LPS-induced sepsis model in HK-2 cells. Overexpression of miR-22-3p remarkably suppresses the inflammatory response and apoptosis throught down-regulating HMGB1, p-p65, TLR4 and pro-inflammatory factors such as IL-1β, IL-6, TNF-α and NO (111). LncRNA Hox transcriptional antisense RNA (HOTAIR) is up-regulated in sepsis-induced renal injury and promotes HK-2 cell apoptosis through HMGB1 (99). Overexpression of miR-381-3p could repress the expression of HMGB1, inhibit apoptosis and inflammatory response, and promote the viability of septic cells (92). Down-regulation of SNHG16 could reduce apoptosis and inflammation in sepsis-induced ALI model by regulating HMGB3 (81). Mitochondrial dysfunction, which is responsible for energy metabolism, intrinsic apoptotic pathway, oxidative stress, and systemic inflammatory responses, is closely related with severe sepsis-induced death (112). *In vitro* and *in vivo*, the researches of HMGNs in the field of apoptosis mainly focus on the tumorigenesis of human cancer and the development of multidrug resistance (113, 114). There is still a lack of sepsis-related research regarding HMGNs.

#### 3.2.2 Necroptosis and HMGs in Sepsis

Necroptosis is programmed death, and is mainly caused by cytokines (TNF-α, IFN-α, IFN-γ), toll-like receptors (TLR3, TLR4, TLR9) and nucleic acid (DNA, RNA) sensors. Receptor interacting serine/threonine protein kinase 1 (RIPK1), receptor inter-acting serine/threonine protein kinase 3 (RIPK3) and mixed lineage kinase domain like pseudokinase (MLKL) are involved, and MLKL is the key molecule. The current research found that necroptosis has been linked to infectious diseases, neurological diseases, inflammatory diseases and cancer (115–118).

In human studies, Yoo et al. demonstrated that plasma HMGB1 was positively correlated with RIPK3 and MLKL, suggesting that HMGB1 is related to necroptosis (119). *In vitro* and *in vivo*, red blood cell (RBC) transfusion enhances the susceptibility to pulmonary inflammation and induces pulmonary endothelial cell necroptosis by releasing HMGB1 (120). The studies of sepsis and HMGs in necroptosis mainly focus on HMGB1, while the studies of other HMGs in necroptosis are still few at present.

#### 3.2.3 Pyroptosis and HMGs in Sepsis

Pyroptosis is inflammatory PCD, which is induced by microbial infection and endogenous injury-related signals (121). Pyroptosis relies on aspartate specific cysteine proteases (Caspase-1, Caspase-4, Caspase-5, Caspase-11) to cleave Gasdermin D (GSDMD) and oligomerize its active N-terminal fragment (GSDMD-NT) in the plasma membrane, then form membrane pores and release proinflammatory factor (IL-1β, IL-18) was the main feature (122). Pyroptosis is one of the natural immune defense mechanisms of the host against pathogen infection (123). According to the different initial activation modes of pyroptosis, which can be divided into three types: the caspase-1-mediated canonical pathway, the caspase-4/5/11-mediated noncanonical pathway, and the pathway of transforming caspase-3-dependent apoptosis into pyroptosis activated by tumor-related chemotherapeutic drugs (124).

LncRNA IGF2-AS regulated nucleotide metabolism by mediating HMGA1 to promote pyroptosis of EPCs in sepsis patients (21). In sepsis mice model induced by CLP, HMGB1 might promote the pyroptosis of liver macrophages and mediate the acute liver injury of sepsis (125). In endotoxemia and bacterial sepsis, HMGB1 released by hepatocytes is necessary for caspase-11 dependent pyroptosis and mortality. In terms of mechanism, HMGB1 released by hepatocytes binds to LPS and is internalized into lysosomes of macrophages and endothelial cells through RAGE. Subsequently, HMGB1 permeates the phospholipid bilayer in a lysosomal acidic environment. This causes LPS to leak into the cytoplasm and activates caspase-11. Loss of hepatocyte HMGB1, inhibition of hepatocyte HMGB1 release, neutralization of extracellular HMGB1 or RAGE deficiency could prevent caspase-11-dependent pyroptosis and death in endotoxemia and bacterial sepsis. These results suggest that the interaction between HMGB1 and LPS mediates the pyroptosis of caspase-11 dependent fatal sepsis (40). There is no report regarding HMGs in pyroptosis except HMGA1 and HMGB1 at present.

### 3.3 HMGs and Drugs in Sepsis

The treatment of sepsis requires different drugs, including anti-inflammatory drugs, anticoagulants, immunomodulatory drugs and herbal medicines. Drugs are the cornerstone of sepsis treatment, often involving a variety of pathways, among which the pathway involving HMGs has gradually become a research hotspot. The deep understanding of the HMGs and sepsis in drug-related research could facilitate the development of effective treatments and preventive methods for sepsis. Studies on HMGs and sepsis involving drugs are shown in **Table 2**.

**Table 2:** Drugs in HMGs and sepsis. Reference given as PMID.

Name	Drug	Type	Disease	Study Model	Summary	Ref (PMID)
HMGB1	indoprofen	anti-inflammatory drug	sepsis	mice	indoprofen exerts a potent therapeutic effect against sepsis by alleviating HMGB1-mediated inflammatory responses.	34755645
HMGB1	glucan phosphate	anti-inflammatory drug	sepsis-induced cardiac dysfunction	mice	glucan phosphate attenuates myocardial HMGB1 translocation in severe sepsis through inhibiting NF-κB activation.	21642503
HMGB1	glucan phosphate	anti-inflammatory drug	sepsis	H9C2 cells	glucan phosphate attenuates HMGB1 release from rat myocardial H9C2 cells in LPS-induced sepsis by inhibiting the activation of NF-κB.	28447579
HMGB1	hydrogen gas	anti-inflammatory drug	sepsis-induced intestinal injuries	mice	2% H2 increases the survival rate, alleviates the injuries caused by oxidative stress and inflammation through reducing HMGB1 levels.	28234792
HMGB1	hydrogen gas	anti-inflammatory drug	sepsis	mice	the beneficial effects of H2 treatment on sepsis and sepsis-associated organ damage are associated with the decreased levels of HMGB1 in serum and tissue.	19997046
HMGB1	xuebijing	herbal medicine	sepsis-induced ALI	mice	xuebijing ameliorates sepsis-induced ALI by down-regulating HMGB1 and RAGE expressions in mice.	25821501
HMGB1	xuebijing	herbal medicine	sepsis	rat	xuebijing exhibits protective efficacy on sepsis by inhibiting the expression of HMGB1.	26313171
HMGB1	ulinastatin	herbal medicine	sepsis-induced ALI	rat	ulinastatin might decrease the lung injury and increase the survival time of ALI rats by downregulating HMGB1 expression.	25966207
HMGB1	reduning	herbal medicine	sepsis	mice	reduning injection protects against sepsis partly *via* inhibition of HMGB1/TLR4/NF-κB/MAPKs signaling pathways.	33421596
HMGB1	glycyrrhizin	herbal medicine	sepsis	rat	glycyrrhizin may protect rats against sepsis by blocking the interaction of HMGB1 with cell surface receptors and HMGB1-mediated inflammatory responses.	28484719
HMGB1	andrographolide	herbal medicine	sepsis	HUVECs/mice	andrographolide inhibits HMGB1-induced inflammatory responses in human umbilical vein endothelial cells and in murine polymicrobial sepsis.	24581270
HMGB1	sesamin	herbal medicine	sepsis	mice	sesamin improves the 7-day survival rate of septic mice, suppresses the inflammatory response in sepsis through the HMGB-1/TLR4/IL-33 signaling pathway.	32893702
HMGB1	emodin	herbal medicine	sepsis-induced ALI	rat/cell	emodin alleviates sepsis-mediated ALI *via* inhibition and reduction of NF-kB and HMGB1 pathways mediated by SIRT1.	34806822
HMGB1	papaverine	herbal medicine	sepsis-induced neuropathy	rat	papaverine’s neuroprotective effects possibly stem from the suppression of the RAGE-HMGB1 axis.	32842806
HMGB1	salidroside	herbal medicine	sepsis-induced ALI	mice	salidroside protects against sepsis-induced ALI and mortality, which might be through the HMGB1 nucleocytoplasmic translocation.	28931916
HMGB1	acteoside	herbal medicine	sepsis	Raw264.7 cells/mice	acteoside reduces HMGB1 release and may be beneficial for the treatment of sepsis.	23563799
HMGB1	toddalolactone	herbal medicine	sepsis	RAW 264.7 cells/mice	toddalolactone protects LPS-induced sepsis and attenuates LPS-induced inflammatory response by modulating HMGB1-NF-κB translocation.	32153412
HMGB1	calycosin	herbal medicine	sepsis-induced ALI	rat	calycosin alleviates sepsis-induced ALI in young rats by inhibiting the HMGB1/MyD88/NF-κB pathway and NLRP3 inflammasome activation.	33857805
HMGB1	naringin	herbal medicine	sepsis	macrophages/mice	naringin decreases TNF-α and HMGB1 release from LPS-stimulated macrophages and improves survival in a CLP-induced sepsis mice.	27716835
HMGB1	quercetin	herbal medicine	sepsis	rat	quercetin exertes protective effects on a rat model of sepsis *via* inhibition of reactive oxygen species (ROS) and downregulation of HMGB1 protein expression.	31377749
HMGB1	cyclopia subternata	herbal medicine	sepsis	HUVECs/mice	vicenin-2 and scolymoside, which are derived from cyclopia subternata, effectively inhibites lipopolysaccharide-induced release of HMGB1, and suppresses HMGB1-mediated septic responses.	26243020
HMGB1	persicarin	herbal medicine	sepsis	HUVECs/mice	persicarin potently inhibits the release of HMGB1 and down-regulates HMGB1-dependent inflammatory responses in human endothelial cells.	22911316
HMGB1	chlorogenic acid	herbal medicine	sepsis	macrophages	chlorogenic acid attenuates HMGB1 and enhances host defense mechanisms in murine sepsis.	23168580
HMGB1	green tea	herbal medicine	sepsis	mice	green tea rescues mice from lethal sepsis partly by inhibiting HMGB1.	17987129
HMGB1	rhododendron brachycarpum	herbal medicine	sepsis	mice	rhododendron brachycarpum may produce a unique scaffold that is developed into a drug mitigating HMGB1-induced vascular pro-inflammation and alleviating severe sepsis and related manifestations.	24576671
HMGB1	cornuside	herbal medicine	sepsis	HUVECs/mice	cornuside suppresses excessive permeability and inhibits HMGB1 release and improves histological conditions in the CLP-induced septic mice model.	35216180
HMGB1	black ginseng	herbal medicine	sepsis	HUVECs/mice	SB1 and SB2, which are derived from black ginsen, reduces the cecal ligation and puncture-induced release of HMGB1, sepsis-related mortality, and tissue injury *in vivo*.	30496780
HMGB1	sodium tanshinone IIA sulfonate	herbal medicine	sepsis-induced cardiac dysfunction	rat	in sepsis, STS efficiently suppresses inflammatory response in myocardium and reduces myocardial necrosis through markedly reducing production of myocardial HMGB1.	30502766
HMGB1	zingerone	herbal medicine	sepsis	HUVECs/mice	zingerone reduces HMGB1-mediated septic responses and improves survival in septic mice.	28610995
HMGB1	sulforaphane	herbal medicine	sepsis	HUVECs/mice	sulforaphane reduces HMGB1-mediated septic responses and improves survival rate in septic mice.	28830206
HMGB1	aloin	herbal medicine	sepsis	HUVECs/mice	aloin reduces HMGB1 release and sepsis-related mortality by activating SIRT1 and PI3K/Nrf2/HO-1 signals.	30966773
HMGB1	baicalein	herbal medicine	sepsis-induced cardiac hypertrophy	rat	baicalein protect cardiomyocytes from LPS-induced cardiac injury through the inhibition of the HMGB1 and MMP-2/-9 signaling pathways.	25004875
HMGB1	green rooibos	herbal medicine	sepsis	HUVECs/mice	aspalathin and nothofagin, which are derived from green rooibos, effectively inhibits lipopolysaccharide (LPS)-induced release of HMGB1.	26224030
HMGB1	maslinic acid	herbal medicine	sepsis	HUVECs/mice	maslinic acid significantly reduces the release of HMGB1 in HUVECs and attenuated the CLP-induced release of HMGB1.	32163831
HMGB1	mung bean	herbal medicine	sepsis	macrophages/mice	mung bean coat extract has protective effect against lethal sepsis possibly by stimulating autophagic HMGB1 degradation.	23193422
HMGB1	angelica sinensis	herbal medicine	sepsis	macrophages/mice	angelica sinensis has protective effect against lethal endotoxemia and experimental sepsis, in part by reducing systemic accumulation of the late pro-inflammatory cytokine HMGB1.	16424112
HMGB1	curcumin	herbal medicine	sepsis	macrophages/mice	curcumin improves the survival of endotoxemic mice by inhibiting nitric oxide-dependent HMGB1 release.	28929026
HMGA1	netropsin	minor-groove binder drug	endotoxaemia	macrophages/mice	netropsin improves survival from endotoxaemia by disrupting HMGA1 binding to the NOS2 promoter.	18937643
HMGB1	biapenem	anti-biotic	sepsis	HUVECs/mice	reduction of HMGB1’s release and septic mortality by biapenem indicate a possibility of successful repositioning of biapenem for the treatment of sepsis.	33515401
HMGB1	cilostazol	anti-coagulant drug	sepsis	RAW 264.7 cells/mice	cilostazol inhibits HMGB1 release in LPS-activated RAW 264.7 cells and increases the survival of septic mice.	26116490
HMGB1	immunoglobulin	immunomodulator	sepsis	rat	high-dose intravenous immunoglobulin reduces the mortality and pulmonary pathology in a rat model of sepsis.	18500418
HMGB1	ketamine	anesthetic drug	sepsis-induced ALI	rat	ketamine protects rats against HMGB1-RAGE activation in a rat model of sepsis-induced ALI, which may partially result from reductions in NF-κB and MAPK.	26945830
HMGB1	ketamine	anesthetic drug	sepsis	macrophages	ketamine inhibits the release of HMGB1 in LPS-stimulated macrophages, and this effect is at least partly mediated by the activation of the Nrf2/HO-1 pathway and NF-κB suppression.	25807407
HMGB1	metformin	anti-diabetic drug	endotoxaemia	RAW 264.7 cells/mice	metformin improves survival in mice model of lethal endotoxaemia by inhibiting HMGB1 release.	21091653
HMGB1	paclitaxel	anti-tumor drug	sepsis-induced AKI	patient/cell	paclitaxel protects against LPS-induced AKI *via* the regulation of lnc-MALAT1/miR-370-3p/HMGB1 axis.	32998017
HMGB1	chloroquine	anti-malarial drug	sepsis	mice	chloroquine inhibits HMGB1 inflammatory signaling and protects mice from lethal sepsis.	23707973
HMGB1	magnesium sulfate	anti-convulsive drug	sepsis-induced diaphragm dysfunction	rat	magnesium sulfate ameliorates sepsis-induced diaphragm dysfunction in rats *via* inhibiting HMGB1/TLR4/NF-κB pathway.	32558672
HMGB1	remifentanil	painkiller	sepsis	rat	remifentanil inhibits expression of HMGB1 in vital organs and release of HMGB1 into plasma.	28145661

HUVECs, human umbilical vein endothelial cells; H2, hydrogen gas; LPS, lipopolysaccharide; CLP, cecal ligation and puncture; STS, Sodium tanshinone IIA sulfonate.

#### 3.3.1 Anti-Inflammation Drugs and HMGs in Sepsis

In recent years, through the continuous research on the pathogenesis of sepsis, researchers have gradually realized that sepsis is a pathophysiological abnormality of the body and its own tissue damage caused by infection, rather than the result of the direct action of bacteria or toxins. The uncontrolled excessive inflammatory response and the accompanying cytokine storm in the early stage of sepsis are the main factors that promote the development of the disease and lead to multiple organ dysfunction and death of patients (126). Therefore, timely and appropriate antagonistic excessive release of inflammatory mediators has become one of the mainstream ideas of drug discovery for the treatment of sepsis. As an important late inflammatory factor, HMGB1 is involved in the pathogenesis of sepsis. Compare with the early inflammatory factors, HMGB1 appears later and lasts longer, and its function is not limited to the simple pro-inflammatory response, but also closely relates to the cellular immune dysfunction of the body, suggesting that HMGB1 intervention may contribute to sepsis (127).

In sepsis mice model induced by CLP, Indoprofen is a non-steroidal anti-inflammatory drug (NSAID), Bi X et al. demonstrated that indoprofen inhibited inflammatory responses and protected mice from lethal organ damages by down-regulating HMGB1 (128). Glucan phosphate (GP) is a carbohydrate ligand that modulates innate immunity and proinflammatory signaling in sepsis (129). GP could inhibit myocardial HMGB1 translocation in severe sepsis and result in attenuation of cardiac dysfunction and improve outcome (130). *In vivo*, GP could attenuate sepsis-induced cardiac dysfunction by inhibiting the release of HMGB-1 (131). Hydrogen gas (H2), which has anti-oxidative, anti-inflammatory, and anti-apoptotic effects (132). Xie K et al. found that the beneficial effects of H2 treatment on sepsis and sepsis-associated organ damage were associated with the decreased levels of oxidative product, increased activities of antioxidant enzymes, and reduced levels of HMGB1 in serum and tissue (133). Similarly, Yu Y et al. also demonstrated that 2% H2 inhalation might be a promising therapeutic strategy for intestinal injuries caused by severe sepsis through the regulation of HO-1 and HMGB1 release (134).

#### 3.3.2 Herbal Medicines and HMGs in Sepsis

Drugs from herbal medicines have great immunomodulatory potential by inhibiting pro-inflammatory and anti-inflammatory cytokines, showing few unnecessary secondary reactions. At the same time, in inflammatory diseases, herbs tend to regulate the level of oxidative stress and inflammation (135). Natural compounds have attracted much attention for the treatment of a variety of clinical complications. Considering that medicinal plants have less side effects and are easy to obtain, comprehensive research on the treatment of sepsis by medicinal plants should be considered.

There are many herbal medicines that have been used as agents in sepsis, including Xuebijing (136, 137) and Ulinastatin (138). Xuebijing is a Chinese patent medicine, which had the advantages of detoxification and toning, elimination of bacteria and viruses, supplementation of vital energy, and improved blood circulation (136, 137). Ulinastatin is an important intrinsic broad-spectrum protease inhibitor, and is generally believed to manage a series of proinflammatory mediators and cytokines (138). Reduning (RDN), a popular traditional Chinese medicine, formulated by three herbs, has been widely used to treat upper respiratory infectious diseases in China (70). Wang Z et al. found that RDN and its effective constituent luteoloside protect against sepsis partly *via* inhibition of HMGB1/TLR4/NF-κB/MAPKs signaling pathways (70). Glycyrrhizin (GL), a natural triterpene glycoside derived from licorice, which protected rats from sepsis by blocking HMGB1 signaling (139). Sesamin is a lignin component isolated from sesame oil and is commonly considered a healthy food with anti-hypertensive, anti-inflammatory, anti-viral and anti-oxidative properties, Li ZL et al. demonstrated that sesamin attenuates intestinal injury in sepsis through the HMGB1/TLR4/IL-33 signaling pathway (140). Quercetin exerts protective effects in a sepsis rat model by quenching ROS, activating of the enzymic antioxidant system, and reducing HMGB1 expression (141). Other herbal medicines, including angelica sinensis (142), maslinic acid (143), baicalein (144), aloin (145) and zingerone (146), play a protective role in sepsis through different mechanisms. The mechanisms of these herbal medicines-related HMGs in sepsis are shown in **Table 2**.

#### 3.3.3 Other Drugs and HMGs in Sepsis

The pathophysiology of sepsis involves infection, coagulation disorder and immunosuppression. Antibiotics, anticoagulant drugs and immunomodulators are effective in the treatment of sepsis. With the deepening of research, drugs for the targeted inhibition of HMGB1 in sepsis have been issued, including painkillers, anti-diabetic drugs and anesthetics (147–149).

Biapenem is an antibiotic that reduces HMGB1 release and septic mortality (150). Cilostazol is anti-platelet agent that inhibits HMGB1’s release in LPS-activated RAW 264.7 cells and increases the survival of septic mice (151). Hagiwara S et al. showed that intravenous immunoglobulin improves pulmonary pathology and overall survival in sepsis by inhibiting the inflammatory response, in particular HMGB1 (152). Ketamine, an intravenous anesthetic agent, has been shown to possess anti-inflammatory effects (153). It attenuates sepsis-induced ALI through HMGB1-RAGE pathways (154), and reduces LPS-induced HMGB1 through the activation of the Nrf2/HO-1 pathway and NF-κB suppression (148). Metformin is one of the most widely prescribed drugs for the treatment of type 2 diabetes (155). It improves the survival in mice model of lethal endotoxaemia by inhibiting HMGB1 release (149). Remifentanil (a painkiller) (147), magnesium sulfate (an anti-convulsive drug) (156), chloroquine (an anti-tumor drug) (157) and paclitaxel (an anti-tumor drug) (91) are involved in HMGs and sepsis-related researches. The mechanisms of drugs described above used in sepsis are shown in **Table 2**.

Sepsis is an extremely complex clinical syndrome, systemic inflammatory response and immune dysfunction are very prominent in the course of clinical manifestation. They often coexist in sepsis pathological process at the same time. The precision of sepsis inflammation and immune regulation make the choose of appropriate drugs great difficult. Prior to entering clinical trials, many sepsis drugs have been shown to improve survival in one or more sepsis animal models. However, when most of these compounds are tested in patients, the improvement in survival is not significant and there is no statistical significance (158, 159). In sepsis, the use of HMGs related drugs also may have the above problems, which need to be solved by researchers.

### 3.4 Other Studies Regarding HMGs in Sepsis

Sepsis is a SIRS caused by infection, which may lead to multiple organ dysfunction. The pathophysiology of sepsis involves inflammatory response and immune response. HMGs act as a potent pro-inflammatory cytokine involved in the delayed endotoxin lethality and systemic inflammatory response (40). HMGs increase in serum and tissues during infection, especially in sepsis. A growing number of studies have demonstrated that HMGs are potential therapeutic targets in the experimental model of sepsis (40). The mechanisms of sepsis are related to HMGs and have aroused extensive interest of researchers.

#### 3.4.1 HMGs in Imbalance Between Pro-Inflammation and Anti-Inflammation

Overexpression of HMGA1 aggravates cardiomyocytes inflammation in sepsis-induced cardiomyopathy (109). HMGB1 is a late proinflammatory cytokine, the circulating HMGB1 level can reflect the disease severity and is potent in augmenting systemic inflammation (160). HMGB1 is an important factor that might link gut bacterial translocation and systemic inflammation in severe acute pancreatitis (161). Comparisons of the necrotic cell debris from HMGB1-deficient and wild-type cells demonstrated that HMGB1-deficient cells had a profoundly reduced capacity to induce cytokines (162). Toddalolactone protectes LPS-induced sepsis and attenuates LPS-induced inflammatory response by modulating HMGB1-NF-κB translocation (163). HMGB1 antibody alters inflammation in murine sepsis model and reduces sepsis mortality (164). ATF3 is a negative regulator of TLR4 signaling, up-regulation of ATF3 contributes to the reduced release of inflammatory molecules (especially HMGB1), which increases the survival rate of mice after LPS challenge (165).

#### 3.4.2 HMGs in Coagulation Disorders

The release and activation of F3 (the main initiator of coagulation) from myeloid or epithelial cells is facilitated by activating inflammasomes and consequent gasdermin D (GSDMD)-mediated pyroptosis, coupled to signal through HMGB1, stimulator of interferon response CGAMP interactor 1 (STING1), or sequestosome 1 (SQSTM1) (166). Extracellular HMGB1 markedly increases the procoagulant activity of tissue factor by promoting the externalization of phosphatidylserine to the outer cell surface. Type 1 interferons (IFNs), a widely expressed family of cytokines that orchestrate innate antiviral and antibacterial immunity, mediates bacterial infection-induced DIC by amplifying the release of HMGB1 into the bloodstream. Inhibition of the expression of type 1 IFNs and disruption of their receptor IFN-α/βR or downstream effector (e.g., HMGB1) uniformly decrease gram-negative bacteria-induced DIC (167).

#### 3.4.3 HMGs in Immunosuppression

HMGB1 can actively affect the immune functions of many types of cells including T lymphocytes, regulatory T cells (Tregs), dendritic cells (DCs), macrophages, and natural killer cells (NK cells). Various cellular responses can be mediated by HMGB1 which binds to cell-surface receptors [e.g., the receptor for advanced glycation end products (RAGE), cTLR2 and TLR4]. Anti-HMGB1 treatment, such as anti-HMGB1 polyclonal or monoclonal antibodies, inhibitors (e.g., ethyl pyruvate) and antagonists (e.g., A box), can protect against sepsis lethality (168). HMGB1 activates the innate immune system and promotes inflammation in conditions such as sepsis (169). HMGB1 regulates intracellular cascades influencing immune cell functions, including chemotaxis and immune modulation (170). HMGB1 secretion is critical for the immunity system because dendritic cells, when reaching the lymph nodes, secrete HMGB1, sustaining the proliferation of antigen-specific T-cells, to prevent their activation-dependent apoptosis, and to promote their polarization toward a T-helper 1 phenotype (171). Targeted expression of a dominant negative HMGA1 transgene is able to improve outcomes in models of endotoxin exposure and microbial sepsis, in part by modulating the immune response (19). Human macrophage and dendritic cell-specific silencing of HMGB1 ameliorates sepsis in a humanized mice model (172). Anti-brain HMGB1 could improve dendritic cell dysfunction in sepsis mice (173).

#### 3.4.4 HMGs in Multiple Organ Dysfunction

In cellular inflammation models, decreasing HMGB1 alleviates LPS-induced sepsis and MODS *in vitro (* 85). The inhibition of cerebral HMGB1 significantly alleviates multiple organ damage under septic exposure, including alleviating the damage to the heart, liver, lungs, and kidneys, which increases the survival rate of septic rats (174). HMGB1 level is an independent risk factor for sepsis and MODS in patient with severe blunt chest trauma (175). HMGB1 is involved in bone marrow mesenchymal stem cells (BMSCs) treatment for MODS, through regulation of the TLR2, TLR4-mediated NF-κB signal pathway (176).

#### 3.4.5 HMGs in Gene Polymorphism

Lee K et al. investigated the associations of a single nucleotide polymorphism (SNP; rs1045411) in HMGB1 with sepsis, and found that the variant A allele of rs1045411 appeared to be associated with a more severe inflammatory response than the GG genotype under specific conditions (177). The rs2249825 SNP and the haplotype TCG are significantly associated with LPS-induced HMGB1 production by peripheral blood leukocytes (178). There are also significant differences in sepsis morbidity rate and MODS scores among patients with different genotypes of the rs2249825. In addition, the patients with the wild-type haplotype TCG have lesser sepsis morbidity rate and MODS scores than those without the TCG haplotype (179).

## 4 Conclusion

Sepsis is an extremely complex clinical syndrome and remains a common and fatal problem worldwide. The discovery of HMGs as an effective cytokine mediator in sepsis has triggered a new field of investigation related to the treatment of sepsis. In the studies about HMGs and non-coding RNAs in sepsis, many miRNA/lncRNA and few circRNA participate in the mechanism of sepsis by targeting HMGs, especially HMGB1. In the studies about HMGs and programmed cell death in sepsis, HMGB1 and HMGA1 induce cell’s apoptosis and pyroptosis, and HMGB1 also induces necroptosis. Systemic inflammatory response and immune dysfunction often coexist in sepsis pathological process at the same time. The precision of sepsis inflammation and immune regulation make the choose of appropriate drugs great difficult. Although many compound drugs which related to sepsis have been shown to improve survival in one or more sepsis animal models, there is no positive effect on the survival improvement in patient. Chinese herbs spark interest in their use as a potential therapeutic agent. In short, HMGs, especially HMGA1 and HMGB1, are widely studied and demonstrated to be involved in the occurrence and development of sepsis. They can not only be used to evaluate the severity of sepsis, but also be potential therapeutic targets of sepsis. Although several lines of evidence have clarified the underlying mechanisms of HMGs in sepsis, the development of an effective treatment that includes HMGs has a long way to go. In order to provide more accurate targeted therapy for sepsis, more detailed studies on HMGs are needed.

## Author Contributions

GL conceived the study, data analysis, and drafted the manuscript. ZH conceived the study, its design and critically revised the manuscript. All authors read and approved the final manuscript.

## Funding

The work was supported by the Natural Science Foundation of Hunan Province (2021JJ31005).

## Conflict of Interest

The authors declare that the research was conducted in the absence of any commercial or financial relationships that could be construed as a potential conflict of interest.

## Publisher’s Note

All claims expressed in this article are solely those of the authors and do not necessarily represent those of their affiliated organizations, or those of the publisher, the editors and the reviewers. Any product that may be evaluated in this article, or claim that may be made by its manufacturer, is not guaranteed or endorsed by the publisher.
