# Supplementary material for: High Mobility Group Proteins in Sepsis
Source: Front Immunol. 2022 Jun 2;13:911152. doi: 10.3389/fimmu.2022.911152 (PMC9202578; doi:10.3389/fimmu.2022.911152)
Supplement: Supplementary file 1 [file Table_1.docx]

| Name | Full name | Structural feature | Function |
| --- | --- | --- | --- |
| MiRNA | Mirco RNA | a single-stranded small RNA molecules with a size of about 21-23 nucleotides, which are generated by 70-90 single-stranded RNA precursors with hairpin structure after processing by Dicer enzyme | participates in almost all types of gene regulation through the following methods: (i) targeting 3 -UTR of mRNA, (ii) argeting 5 -UTR of mRNA, (iii) targeting coding region of mRNA, and (iv) embedding in a specific gene. |
| LncRNA | Long non-coding RNA | a subtype of RNA transcripts which contain more than 200 nucleotides, lacking in capability of encoding protein or exhibiting limited potential | epigenetic regulation, transcriptional regulation, post-transcriptional regulation, regulation of miRNA |
| CircRNA | Circular RNA | a non-coding RNAs with closed ring structure, without 5' cap structure and 3'poly (A) structure, which is not affected by RNA exonuclization | miRNA sponge, regulatory protein binding, regulatory gene transcription and coding |

**Table S1 : Structuralfeatures and function of miRNA, lncRNA, circRNA**
