# Supplementary material for: High Mobility Group Proteins in Sepsis
Source: Front Immunol. 2022 Jun 2;13:911152. doi: 10.3389/fimmu.2022.911152 (PMC9202578; doi:10.3389/fimmu.2022.911152)
Supplement: Supplementary file 2 [file Table_2.docx]

|  | **Apoptosis** | | | | **Necroptosis** | **Pyroptosis** | | |
| --- | --- | --- | --- | --- | --- | --- | --- | --- |
| **Concept** | Active cell controlled death processes that do not leak their contents into the surrounding environment | | | | Passive uncontrolled cell death, spilling contents into the surrounding environment, leads to inflammation and tissue damage | An active process of controlled cell death in which contents and inflammatory factors are released into the surrounding environment | | |
| **Origin** | Cells sense their own damage; the interaction of damaged cells with immune cells | | | | Cells are severely damaged by sudden extreme stimulation, such as hypoxia and malnutrition | pathogen | | |
| **Caspase** | Starting caspases: caspase 8, 9, 10  Perform caspases: caspase 3, 6, 7 | | | | —— | Caspase 1, 3, 4, 5, 11 | | |
| **Landmark event** | Nuclear fragmentation, plasma membrane blebbing, cell shrinkage (pyknosis), formation of apoptotic bodies  and phagocytosis by neighbouring cells.  Pro-apoptotic BCL-2 family members, caspase activation, cleavage of hundreds of caspase substrates | | | | Nuclear fragmentation, plasma membrane blebbing, cell shrinkage (pyknosis), formation of apoptotic bodies  and phagocytosis by neighbouring cells.  Pro-apoptotic BCL-2 family members, caspase activation, cleavage of hundreds of caspase substrates | Rupture of the plasma membrane and lack of cell swelling. Inflammatory induced activation of the initiator  caspases, caspase-1 and -11, and consequent activation of the effector caspases, caspase-3 and -1. Release of  bio-active IL-1β and IL-18 and proteolytic activation of GSDMD, the essential effector of pyroptosis. | | |
| **Classification** | extrinsic or death receptor pathway | intrinsic or mitochondrial pathway | intrinsic endoplasmic reticulum pathway | perforin/granzyme pathway | —— | caspase-1-mediated canonical pathway | caspase-4/5/11-mediated noncanonical pathway | the pathway of transforming caspase-3-dependent apoptosis into pyroptosis |

**Table S2 : Characteristics of apoptosis, necroptosis and pyroptosis**
